# Urocortin ameliorates diabetic cardiomyopathy in rats via the Akt/GSK-3β signaling pathway

**DOI:** 10.3892/etm.2015.2211

**Published:** 2015-01-23

**Authors:** XINYU LIU, CHUNNA LIU, XIAOYAN ZHANG, JIAJUN ZHAO, JIN XU

**Affiliations:** 1 Department of Endocrinology, Provincial Hospital Affiliated to Shandong University, Jinan, Shandong 250021, P.R. China; 2 Department of Endocrinology, The First Affiliated Hospital of Liaoning Medical University, Jinzhou, Liaoning 121001, P.R. China; 3 Department of Pharmacology, Liaoning Medical University, Jinzhou, Liaoning 121001, P.R. China

**Keywords:** urocortin, diabetic cardiomyopathy, cardiac fibrosis, Akt/glycogen synthase kinase-3β, creatine phosphokinase isoenzyme, transforming growth factor-β1

## Abstract

Urocortin has been shown to exert powerful protective effects on various cardiovascular disease models. However, the role and mechanism of urocortin in protecting against diabetic cardiomyopathy (DCM) has not yet been elucidated. In the present study, the effects of urocortin on cardiac dysfunction, fibrosis, inflammation and the interrelated signaling pathways were investigated in a diabetic rat model. Diabetes mellitus (DM) was induced in the rats by intraperitoneal injection of streptozotocin. The diabetic rats were randomly divided into four groups: Diabetic control, urocortin, urocortin + astressin treatment and urocortin + triciribine treatment groups. All the experiments were conducted at 16 weeks following the induction of DM. The levels of glycosylated hemoglobin (HbA1c), creatine phosphokinase isoenzyme (CK-MB) and plasma brain natriuretic peptide (BNP), as well as the myocardial collagen volume fraction (CVF) and left ventricular mass index (LVWI), were measured. In addition, levels of inflammatory factors, including transforming growth factor (TGF)-β1, connective tissue growth factor (CTGF) and interrelated proteins, such as Akt and glycogen synthase kinase (GSK)-3β, were detected by biochemical analyses. In the diabetic group, the levels of BNP and CK-MB, as well as the mRNA and protein expression levels of TGF-β1 and CTGF, and the LVWI and CVF, were higher compared with the rats in the control group (P<0.05). This was accompanied by decreased Akt and GSK-3β phosphorylation (P<0.05). Notably, urocortin attenuated myocardial dysfunction, cardiac fibrosis and inflammation in the hearts of the diabetic rats. However, urocortin exhibited no effect on the level of HbA1c. In addition, the inhibited phosphorylation of Akt and GSK-3β was restored with urocortin administration. However, all the effects of urocortin were eliminated with treatment of the corticotropin releasing factor receptor 2 antagonist, astressin. Triciribine, an Akt inhibitor, partially eliminated the effects of urocortin on myocardial dysfunction, inflammation and cardiac fibrosis in the hearts of the diabetic rats. These results indicated that urocortin may exhibit great therapeutic potential in the treatment of DCM by attenuating fibrosis and inflammation. Furthermore, the Akt/GSK-3β signaling pathway may be partially involved in mediating these effects.

## Introduction

Cardiomyopathy, a severely disabling complication of diabetes mellitus (DM), is the leading cause of mortality among adults worldwide (1). Individuals with cardiomyopathy are often at a risk of suffering from an irregular heart beat and sudden cardiac mortality. Although the cause of cardiomyopathy is poorly understood, the pathophysiology of diabetic cardiomyopathy (DCM) is hypothesized to be multifactorial (2). In DM, the myocardial tissue structure changes and dysfunction that are induced by factors other than coronary artery disease and cardiac neuropathy are defined as DCM (3).

The first clinical manifestation of DCM is diastolic dysfunction, which may be later accompanied by systolic dysfunction. Myocardial cell degeneration and necrosis are the major factors causing pathophysiological changes in DCM (4). Transforming growth factor (TGF)-β1 and connective tissue growth factor (CTGF) are highly expressed in experimental DM hearts, which is in association with cell proliferation, recognition, apoptosis, special differentiation and extracellular matrix accumulation. At present, a number of studies have shown that levels of TGF-β1 and CTGF increase in myocardial tissues of diabetic patients, thus, these factors are expected to become new targets for the treatment of DCM (5,6).

Urocortin was first described by Vaughan *et al* as a 40-amino-acid peptide associated with the corticotrophin-releasing factor (CRF) family, which binds to and activates type 1 and 2 CRF receptors (CRFRs) (7). Urocortin is distributed in the central nervous system and periphery, in sites such as the Edinger-Westphal nucleus, adipose tissue, heart, kidney and immunological tissue (8). Endothelial urocortin has been shown to suppress the generation of angiotensin II-induced reactive oxygen species production in endothelial cells (9). Urocortin-induced endothelium-dependent relaxation of rat arteries has also been reported (10). In addition, this peptide has been found in the heart and been shown to cause marked vasodilatation of the aorta (11). Administration of urocortin for four days was shown to have sustained beneficial hemodynamics, hormonal and renal effects in an experimental heart failure model (12). A previous study demonstrated that urocortin may play a protective role in ischemia-reperfusion injury in rat hearts against oxidative stress by inhibiting the activities of free radicals (13). Urocortin was also found to exhibit an inhibitory effect on the activity of serum angiotensin converting enzyme (14). Therefore, the results of these studies strongly indicate that urocortin may have a beneficial effect on DCM.

To the best of our knowledge, the underlying mechanisms of urocortin in DCM remain unclear. We hypothesized that DCM may be reversed by urocortin. Thus, in the present study, the role of urocortin in the progression of DCM and the relevant mechanisms involving the Akt/glycogen synthase kinase-3β signaling pathway were investigated. The levels of glycosylated hemoglobin (HbA1c), creatine phosphokinase isoenzyme (CK-MB), brain natriuretic peptide (BNP), TGF-β1 and CTGF, as well as the collagen volume fraction (CVF) and left ventricular mass index (LVWI), were used to estimate the effect of urocortin on DCM, mediated by the CRFR-2.

## Materials and methods

### Animals and supplementation

Animal care and experimental protocols were carried out in accordance with the Guide for the Care and Use of Laboratory Animals (NIH publication no. 85-23, revised 1996) and approved by the Ethics Committee of Shandong University (Jinan, China). A total of 50 male Wistar rats (weight, 250–300 g; age, 18–20 weeks) were purchased from the Experimental Animal Center of Shanghai Animal Institute (Shanghai, China) and used in the study.

DM was induced in 40 rats via intraperitoneal injection of 55 mg/kg streptozotocin (STZ; Sigma-Aldrich, St. Louis, MO, USA) dissolved in 0.1 M citrate buffer (pH 4.5). The ten remaining animals were treated with a vehicle and were referred to as the control group. After three days of STZ injections, the blood glucose levels were measured using a glucometer (AccuCheck; Roche Diagnostics, Mannheim, Germany). Rats that had blood sugar levels of 200 mg/dl, were used for the study. The diabetic rats were divided into four groups (10 animals per group), which included the diabetic (DM), urocortin-treated (UCN; Sigma-Aldrich), urocortin + astressin (UCN + AST) and urocortin + triciribine groups (UCN + TRI). Astressin (Sigma-Aldrich) was used as a CRFR-2 antagonist, while triciribine was used as an inhibitor of Akt. No statistically significant differences in the non-fasting blood glucose levels were observed among the four groups. Rats in the UCN group received 7 μg/kg urocortin intraperitoneally per day; rats in UCN +AST group received 7 μg/kg urocortin and 35 μg/kg astressin intraperitoneally per day; and rats in the UCN + TRI group received 7 μg/kg urocortin and 0.5 mg/kg triciribine intraperitoneally per day for 16 weeks. Rats in the diabetic group received the same volume of normal saline. Age-matched male Wistar rats were used as normal controls (10 animals). The normal control and diabetic rats were housed in a room with a 12-h artificial light cycle with free access to an 18% high-fat diet (standard rat diet, 8% fat) and water. The body weight (BW) and non-fasting blood glucose levels were measured weekly. After 16 weeks of supplementation, the animals were anaesthetized with 1 g/kg^−1^ intraperitoneal urethane and sacrificed. A blood sample was taken from the heart and the serum was isolated for biochemical measurements. The heart was removed under aseptic conditions and perfused with ice-cold diethylpyrocarbonate-treated distilled water (Wuhan Boster Biological Technology, Ltd., Wuhan, China).

### Measurements of BW, LVW and LVWI

After the rats were weighed and anesthetized with intraperitoneal urethane (1 g/kg), the thorax was rapidly opened and the heart was excised. The heart was washed in 0.01 mol/l phosphate-buffered saline and the LVW was detected, from which the LVWI was calculated as LVW/BW.

### Measurements of HbA1c, CK-MB and BNP

CK-MB and BNP levels in the rats were measured using a RA-50 semi-auto analyzer, while HbA1c analysis was performed using a Nycocard Reader (Axis-Shield, Oslo, Norway).

### Myocardial pathology and CVF observations

Heart tissues were paraffin-embedded, cut into sections and stained with hemotoxylin and eosin. In addition, collagen in the heart was specifically stained with Ponceau S and the CVF was determined. In brief, images of the sections were captured and analyzed with Image-Pro Plus 6.0 image analysis software. Five fields were randomly selected and the CVF was calculated as the collagen area/total area, which was followed by averaging. However, the area of collagen surrounding the vessels was not included in the CVF. Four arterioles in the ventricular wall were selected and the cross-sectional area was measured.

### Measurement of TGF-β1 and CTGF in the plasma

Plasma TGF-β1 and CTGF levels were determined using a sandwich ELISA method with a commercially available kit (BD Opt-EIA; BD Biosciences, Franklin Lakes, NJ, USA). Appropriate controls and standards were used, as specified by the manufacturer’s instructions, and the data are expressed as pg/ml of plasma.

### Quantitative polymerase chain reaction (PCR) analysis of TGF-β1 and CTGF mRNA expression

Total RNA was isolated using TRIzol reagent and purified with an RNeasy kit (Qiagen, Valencia, CA, USA), according to the manufacturer’s instructions. Reverse transcription of 500 ng total RNA was performed in a total volume of 20 μl using an iScript cDNA synthesis kit (Bio-Rad, Hercules, CA, USA). In total, 1 μl cDNA was amplified by PCR in 20-μl reactions containing special primers and iQ SYBR Green Supermix (Bio-Rad). PCR was performed for 40 cycles, consisting of 95°C for 15 sec, 94°C for 5 sec, 58°C for 15 sec and 72°C for 15 sec, using the iCycle iQ Real-Time PCR Detection System (Bio-Rad). The primers used for PCR were purchased from Invitrogen Life Technologies (Grand Island, NY, USA) and had the following sequences: TGF-β1 forward, 5′-GCTCGCTTTGTACAACAGCA-3′ and reverse, 5′-GAGTTCTACGTGTTGCTCCA-3′; β-actin forward, 5′-CCTCTATGCCAACACAGTGC-3′ and reverse, 5′-GTACTCCTGCTTGCTGATCC-3′; CTGF forward, 5′-CAAGGGCCTCTTCTGTGACT-3′ and reverse, 5′-TGGAGATTTTGGGAGTACGG-3′; β-actin forward, 5′-TGACGGGGTCACCCACACTGTGCCCATCTA-3′ and reverse, 5′-CTAGAAGCATTTGCGGTGGACGATGGA GGG-3′. Relative mRNA expression levels were determined using the comparative Ct method with data normalized against 36B4 riboprotein mRNA and calibrated to the average ΔCt value of the untreated controls. Data are expressed as a percentage of the control, which was set to 100%.

### Western blot analysis

Proteins were extracted from the heart homogenates, and aliquots (50 μg) were subjected to SDS-PAGE (7.5% gel) and transferred to nitrocellulose membranes. The membranes were blocked for 2 h at room temperature with block solution that was provided in the enhanced chemiluminescence (ECL) kit. The membranes were then incubated with primary antibodies overnight at 4°C. Equal protein samples were used for western blot analysis with the following rat antibodies targeted against: TGF-β1 (EM010-48), CTGF (BC087839), phospho-Akt (Ser 473; GM-AT7126), Akt, phospho-GSK-3β (Ser 9; 07835), GSK-3β (bc000251) and β-actin (all purchased from Biogot Technology Co., Ltd., Shanghai, China). The membranes were then washed for 30 min in wash solution (ECL kit) and incubated with rat secondary IgG antibodies conjugated with horseradish peroxidase in block solution (Biogot Technology Co., Ltd.). The membranes were washed for 30 min in wash solution and the immunoreactive bands were detected with an ECL kit.

### Statistical analysis

Statistical analysis was performed using SPSS version 14.0 statistical software (SPSS, Inc., Chicago, IL, USA) and data are expressed as the mean ± standard deviation. Comparisons of the mean values between multiple groups were performed with one-way analysis of variance, where P<0.05 was considered to indicate a statistically significant difference.

## Results

### Urocortin decreases the LVWI in diabetic rats

**Figure 1:**
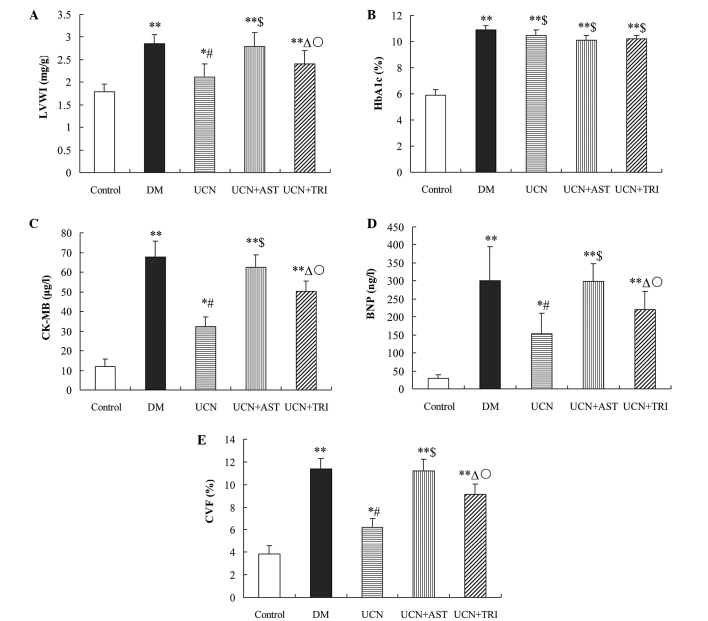
Effect of urocortin on (A) LVWI, (B) HbA1c, (C) CK-MB, (D) BNP and (E) CVF and in the diabetic rats. Levels of BNP and CK-MB, as well as the LVWI and CVF, were decreased by urocortin and urocortin and triciribine treatment, while astressin blunted the effect of urocortin. HbA1c levels were not affected. ^*^P<0.05 and ^**^P<0.01, vs. control; ^#^P<0.01 and ^Δ^P<0.05, vs. DM group and UCN + AST; ^$^P>0.05, vs. DM group; ^○^P<0.05, vs. UCN group. LVWI, left ventricular mass index; HbAlc, glycosylated hemoglobin; CK-MB, creatine kinase isoenzyme; BNP, brain natriuretic peptide; CVF, collagen fraction volume; DM, diabetes mellutis; UCN, urocortin; AST, astressin.

Compared with the control group, a significant increase in the LVWI was observed in the DM (P<0.01), UCN (P<0.05), UCN + AST (P<0.01) and UCN + TRI (P<0.01) groups. Notably, urocortin decreased the LVWI in diabetic rats (P<0.01), and the LVWI was partially decreased by urocortin and triciribine treatment as compared with urocortin treatment alone (P<0.05). However, administering astressin with urocortin appeared to reduce the beneficial effect of urocortin on the LVWI (Fig. 1A).

### Urocortin decreases the levels of CK-MB and BNP

HbA1c, CK-MB and BNP levels in the DM (P<0.01), UCN (P<0.05), UCN + AST (P<0.01) and UCN + TRI (P<0.01) groups were significantly increased compared with the control. No statistically significant differences were observed in the HbA1c levels (P>0.05) among the DM, UCN, UCN + AST and UCN + TRI groups. Compared with the control, the CK-MB and BNP levels in the diabetic rats markedly increased (P<0.01), however, this increase was diminished (P<0.01) by treatment with urocortin and was partially reduced (P<0.05) by urocortin and triciribine treatment when compared with urocortin administration alone. This effect of urocortin on CK-MB and BNP was significantly reversed by astressin (Fig. 1B–D).

### Myocardial CVF inhibition by urocortin

**Figure 2:**
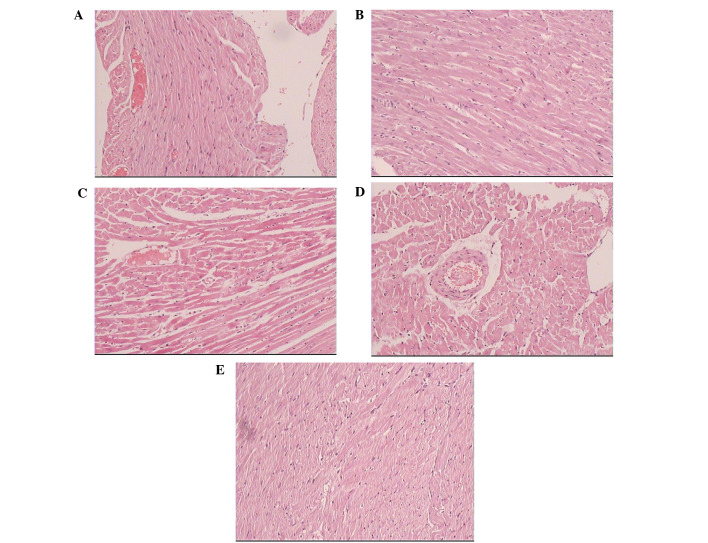
Urocortin alleviated myocardial fibrosis in DCM (HE stain; magnification, ×400). (A) Control; (B) DM; (C) UCN; and (D) UCN + AST and (E) UCN + TRI groups. DCM, diabetic cardiomyopathy; HE, hematoxylin and eosin; DM, diabetes mellitus; UCN, urocortin; AST, astressin.

Cardiomyocytes in the normal rats presented with a regular arrangement, clear stripes without myofilament fracture and uniform intercellular space. By contrast, cardiomyocytes in the diabetic group exhibited myofibrillar disarray and myocardial fibrosis, scattered muscle fiber-degeneration, coarse granules in the cytoplasm, nucleus swelling and deformation, loss of cardiac muscle fiber stripes, interstitial edema and hyperplasia and lymphocytic infiltration. The myocardial cell arrangement in the diabetic rats treated with urocortin was more regular than that in the diabetic group (Fig. 2). Compared with the control group, the CVF in the DM, UCN, UCN + AST and UCN + TRI groups significantly increased (P<0.05). However, urocortin treatment suppressed the CVF increase (P<0.01) in the diabetic rats, while urocortin and triciribine treatment exhibited a partial effect on the CVF (P<0.05). The effect of urocortin was reversed by astressin (Fig. 1E).

### Urocortin decreases the levels of TGF-β1 and CTGF in the plasma of diabetic rats

**Figure 3:**
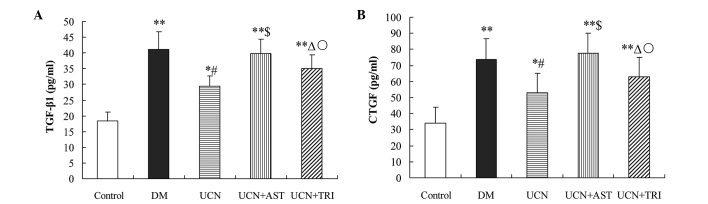
Urocortin and urocortin with triciribine reduced the oversecretion of (A) TGF-β1 and (B) CTGF in the plasma of diabetic rats. However, astressin reversed the effect of urocortin. ^*^P<0.05 and ^**^P<0.01, vs. control; ^#^P<0.01 and ^Δ^P<0.05, vs. DM and UCN + AST groups; ^$^P>0.05, vs. DM group; ^○^P<0.05, vs. UCN group. TGF, transforming growth factor; CTGF, connective tissue growth factor; DM, diabetes mellitus; UCN, urocortin; AST, astressin.

Hyperglycemia is known to activate several cytokines via oxidative stress, which may contribute to the development of DCM. Compared with the control, TGF-β1 and CTGF levels increased significantly (P<0.01) in the plasma of the diabetic rats. Supplementation with urocortin markedly reduced the levels of TGF-β1 and CTGF as compared with DM treatment (P<0.01), while supplementation with urocortin and triciribine partially reduced the TGF-β1 and CTGF levels when compared with urocortin treatment alone (P<0.05). Astressin with urocortin treatment in the diabetic rats appeared to reverse the effect of urocortin on the levels of TGF-β1 and CTGF (Fig. 3).

### Urocortin decreases the expression of TGF-β1 and CTGF in diabetic rats

**Figure 4:**
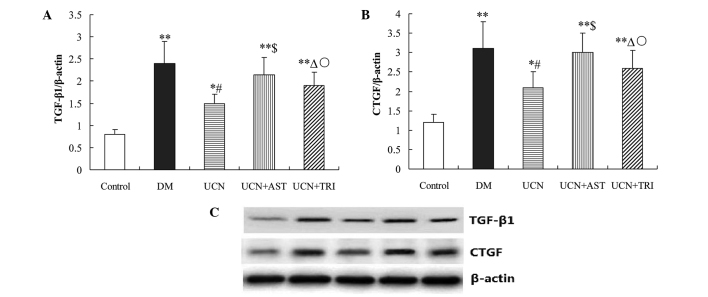
Urocortin and urocortin with triciribine treatment inhibited TGF-β1 and CTGF expression in the cardiac muscular tissues of diabetic rats, while astressin administration eliminated the effects of urocortin. mRNA expression levels of (A) TGF-β1 and (B) CTGF in the various groups as detected by quantitative PCR. (C) Protein expression levels of TGF-β1 and CTGF in the various groups, as detected by western blot analysis. ^*^P<0.05 and ^**^P<0.01, vs. control; ^#^P<0.01 and ^Δ^P<0.05, vs. DM and UCN + AST groups; ^$^P>0.05, vs. DM group; ^○^P<0.05, vs. UCN group. TGF, transforming growth factor; CTGF, connective tissue growth factor; PCR, polymerase chain reaction; DM, diabetes mellitus; UCN, urocortin; AST, astressin.

To investigate the mechanism by which urocortin exhibits beneficial effects on myocardial fibrosis in diabetic rats, the expression levels of TGF-β1 and CTGF were analyzed, since they had been shown to be involved in myocardial fibrosis. The effect of urocortin on TGF-β1 and CTGF expression was investigated in cardiac muscular tissues obtained from each group. Quantitative PCR analysis revealed that TGF-β1 and CTGF mRNA expression levels in the DM (P<0.01), UCN (P<0.05), UCN + AST (P<0.01) and UCN + TRI (P<0.01) groups were higher compared with the control animals. However, urocortin decreased the TGF-β1 and CTGF mRNA expression levels in diabetic rats (P<0.01), and supplementation with urocortin and triciribine partially decreased the TGF-β1 and CTGF mRNA expression levels when compared with urocortin treatment alone (P<0.05). Furthermore, western blot analysis demonstrated that urocortin decreased the protein expression levels of TGF-β1 and CTGF in diabetic rats (P<0.01). However, astressin with urocortin treatment completely eliminated the inhibitory effect of urocortin on TGF-β1 and CTGF overexpression (Fig. 4).

### Urocortin activates the Akt/GSK-3β signaling pathway

**Figure 5:**
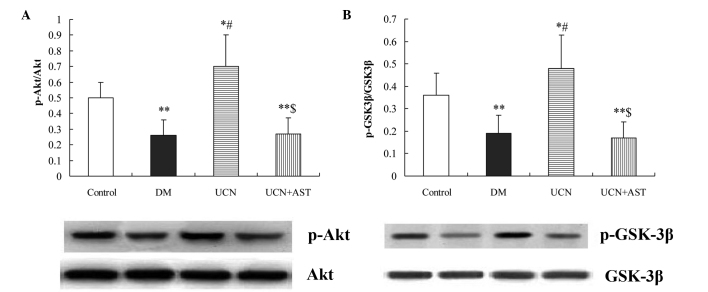
Urocortin activated Akt and inactivated GSK-3β in the cardiac muscular tissues of diabetic rats, while astressin eliminated the effects of urocortin. Western blot analysis of (A) p-Akt and Akt and (B) p-GSK-3β and GSK-3β. ^*^P<0.05 and ^**^P<0.01, vs. control; ^#^P<0.01, vs. DM and UCN + AST groups; ^$^P>0.05, vs. DM group. GSK, glycogen synthase kinase; p-Akt, phospho-Akt; p-GSK, phospho-glycogen synthase kinase; DM, diabetes mellitus; UCN, urocortin; AST, astressin.

Using western blot analysis, Akt phosphorylation was shown to be significantly inhibited in the diabetic hearts. In addition, marked activation of GSK-3β was observed in the diabetic hearts. By contrast, urocortin induced a significant increase (P<0.01) in the phosphorylation of Akt and GSK-3β in the myocardium, while astressin inhibited the effect of urocortin on Akt and GSK-3β phosphorylation (Fig. 5).

## Discussion

DCM is an important cardiovascular complication that causes cardiac dysfunction in DM patients. The main pathological changes include myocardial cell focal hypertrophy, degeneration, necrosis, apoptosis and myocardial remodeling. A series of pathophysiological changes caused by myocardial interstitial remodeling play an important role in the pathogenesis of DCM (15).

The major novel observation of the present study was that urocortin may be beneficial in reversing the effects of DCM. Consistent with pervious studies, the 16-week untreated diabetic rats in the study were characterized by excess collagen accumulation and cardiac hypertrophy. In the untreated diabetic hearts, increased BNP and CK-MB accumulation, coupled with elevated LVWIs and CVFs, were observed. In addition, enhanced myocardial expression of TGF-β1 and CTGF, coupled with inactivated Akt/GSK-3β signaling, were observed, which eventually culminated in cardiac fibrosis. By contrast, urocortin was found to prevent the development of these characteristic alterations of DCM, and these beneficial effects involved CRFR-2. The underlying mechanisms also involved the inhibitory effects of urocortin on the overexpression and secretion of TGF-β1 and CTGF via the activation of the Akt/GSK-3β signaling pathway in the diabetic hearts.

In response to high levels of glucose, the expression of the potent profibrotic factor, TGF-β1, significantly increases, which leads to fibrotic consequences. TGF-β1 is an important fibrogenic factor that promotes the synthesis and secretion of collagen I and III which induces myocardial fibrosis (16). In the development of extracellular matrix accumulation, CTGF may function as a downsteam mediator of TGF-β1 (17). In the present study, LVWI, BNP, CK-MB and CVF levels, as well as TGF-β1 and CTGF mRNA and protein expression levels, were significantly higher in the diabetic group when compared with the control group, indicating that the increase in TGF-β1 and CTGF expression levels in the DM rats was closely associated with myocardial remodeling.

Akt promotes cell survival by inhibiting several targets that are involved in apoptotic signaling cascades. GSK-3β, a major substrate of Akt, not only has central functions in glycogen metabolism and insulin function, but also plays a crucial role in transmitting apoptotic signals, DM-induced inflammation and fibrosis (18). A previous study demonstrated that the activation of GSK-3β played a pivotal role in DM-induced energy metabolic derangement and, consequently, pathological remodeling in the heart (19). In DM, Akt phosphorylation can be reduced by the elevated circulation of free fatty acids and inflammatory cytokines, which leads to the activation of GSK-3β (20). In addition, Akt has a clearly defined role in the regulation of cardiovascular functions, including cardiac growth, contractile function and coronary angiogenesis (21). The results of the present study demonstrated that triciribine, an inhibitor of Akt, partially weakened the effects of urocortin on DCM and the Akt/GSK-β pathways involved in mediating the effects of urocortin on DCM.

Urocortins are endogenous vasoactive peptides that have been shown to exert powerful beneficial neurohormonal, hemodynamic and renal effects in an experimental heart failure model (22). Urocortins function predominantly through two receptor subtypes, CRFR-1 and CRFR-2. The receptors possess seven transmembrane domains and are G-protein coupled. CRF-2(a) receptors constitute the dominant peripheral CRFR-2 form, particularly in the heart and vasculature. Receptor concentrations are high in the left ventricle and intramyocardial vessels (23). However, a recent study revealed that the CRF-2 receptor exhibits much more potent effects on the cardiovascular system compared with CRF (24). The CRF-2 receptor enhances cardiac contractility, coronary blood flow, heart rates and cardiac output. A number of *in vitro* and *ex vivo* investigations have shown that in cases of ischemia/reperfusion, atherosclerosis and hypertension, urocortin can protect cardiac cells from severe injury (25). As a novel small molecular active peptide, urocortin exerts protective effects via autocrine and/or paracrine signaling pathways (26,27). The majority of studies have reported that urocortin binds to CRFR-2 and promotes an increase in cAMP levels, thus, activating protein kinase A (28,29). Brar *et al* found that the mitogen-activated protein kinase and phosphatidylinositol 3-kinase pathways were involved in the protective mechanisms of urocortin (30,31). In a recent study, urocortin was shown to directly activate AMP-activated protein kinase in *ex vivo*-perfused mouse hearts and decrease injury and contractile dysfunction during ischemia/reperfusion. In addition, the study revealed that stimulation of CRFR-2 by CRF and urocortin induced the release of BNP, however, this had an inotropic effect on the heart, which is consistent with the observations of the present study (32).

Considering the role of myocardial interstitial remodeling in diabetic development, the present study aimed to investigate the beneficial effects of urocortin on DCM remodeling. Firstly, it was found that 16 weeks of intervention with urocortin in diabetic rats significantly reduced the levels of BNP and CK-MB, the LVWI and myocardial CVF, as well as the mRNA and protein expression levels of TGF-β1 and CTGF. Secondly, urocortin was shown to significantly promote the phosphorylation of Akt and GSK-3β in the myocardium. However, in the UCN + AST group, the effects of urocortin were prevented, which indicated that the effects were closely associated with the CRFRs. Thirdly, triciribine partially eliminated the effects of urocortin on DCM, indicating that the Akt/GSK-β signaling pathways were partially involved in mediating the effects of urocortin.

In conclusion, the results of the present study support the hypothesis that urocortin markedly inhibits the development of cardiac dysfunction, myocardial fibrosis and inflammation in diabetic rats. Urocortin was shown to protect cardiac functions, reverse ventricular remodeling, inhibit hypertrophy and decrease the collagen content. The predominant underlying mechanism may be that urocortin binds to CRFR-2, which then inhibits the expression of TGF-β1 and CTGF. The Akt/GSK-β signaling pathways are partially involved in mediating the effects of urocortin. These observations provide new guidance for the clinical treatment of DCM. However, it is not known whether these observations have a bearing on the potential clinical application of urocortin as a novel agent, with a potent protective function in the treatment of human DM. In addition, whether alterations in these control pathways contribute to the development of cardiovascular disease remains unknown.

The present study has demonstrated that urocortin significantly improves cardiac function. Urocortin inhibits myocardial fibrosis and inflammation in diabetic rats, which is associated with the inhibition of TGF-β1 and CTGF expression and the Akt/GSK-β signaling pathways. These results provide new guidance for the clinical treatment of DCM.
